# Treatment guided by cerebral oximetry in newborns receiving invasive mechanical ventilation: study protocol for step one of the SafeBoosC-IIIv randomised clinical trial

**DOI:** 10.1186/s13063-026-09631-5

**Published:** 2026-03-17

**Authors:** Caroline Barkholt Kamp, Johanne Juul Petersen, Mathias Lühr Hansen, Adelina Pellicer, Gunnar Naulaers, Eugene Dempsey, Gitte Holst Hahn, Markus Harboe Olsen, Marie Isabel Skov Rasmussen, Gerhard Pichler, Gabriel Dimitriou, Tomasz Szczapa, Maria Livia Ognean, Saudamini Nesargi, Gabriel Musante, Lina Chalak, Massimo Di Maio, Jyoti Lakhwani, Renato S. Procianoy, Jakub Tkaczyk, Hans Fuchs, Merih Cetinkaya, Cornelia Hagmann, Himanshu Popat, Jorge Fabres, Laishuan Wang, Georg Schmölzer, Monica Fumagalli, Salvador Piris-Borregas, Ramona Mohora, Pamela Zafra, Kosmas Sarafidis, Miguel Alsina-Casanova, Nariae Baik-Schneditz, Laura Serrano Lopez, Elke Griesmaier, Eleftheria Hatzidaki, Shashidhar A, Theodore Dassios, Gorm Greisen, Janus Christian Jakobsen

**Affiliations:** 1 Copenhagen Trial Unit, Centre for Clinical Intervention Research, The Capital Region of Denmark, Copenhagen University Hospital – Rigshospitalet, Copenhagen, Denmark; 2 Department of Neonatology, Copenhagen University Hospital – Rigshospitalet, Copenhagen, Denmark; 3 Department of Neonatology, La Paz University Hospital, Hospital La Paz Institute for Health Research-IdIPAZ, Madrid, Spain; 4 University Hospital Leuven, Louvain, Belgium; 5 Infant Research Centre, University College Cork, Cork, Ireland; 6 Department of Neuroanaesthesiology, Neuroscience Centre, Copenhagen University Hospital – Rigshospitalet, The Capital Region, Copenhagen, Denmark; 7 Division of Neonatology, Department of Pediatrics and Adolescence Medicine, Medical University Graz, Graz, Austria; 8 Department of Paediatrics, University of Patras, Patras, Greece; 9 II Department of Neonatology, Neonatal Biophysical Monitoring and Cardiopulmonary Therapies Research Unit, Poznan University of Medical Sciences, Poznan, Poland; 10 Faculty of Medicine, Lucian Blaga University Sibiu, Sibiu, Romania; 11 Department of Neonatology, Clinical County Emergency Hospital Sibiu, Sibiu, Romania; 12 John’s Medical College Hospital, Bengaluru, Karnataka India; 13 Hospital Universitario Austral, Pilar, Argentina; 14 UT Southwestern Medical Center, Dallas, USA; 15 CHU Nîmes, Nimes, France; 16 Women and Newborn Hospital, University Teaching Hospitals, Lusaka, Zambia; 17 Hospital de Clínicas de Porto Alegre, Universidade Federal Do Rio Grande Do Sul, Porto Alegre, Brazil; 18 University Hospital Motol, Prague, Czech Republic; 19 Medical Center, University of Freiburg, Freiburg, Germany; 20 Department of Neonatology, Health Sciences University, Basaksehir Cam and Sakura City Hospital, Istanbul, Turkey; 21 Department of Neonatology and Intensive Care, University Children’s Hospital Zürich, Zurich, Switzerland; 22 Grace Centre for Newborn Intensive Care in The Children’s Hospital at Westmead, Sydney, Australia; 23 NHMRC Clinical Trials Centre, University of Sydney, Sydney, Australia; 24 Department of Pediatrics, School of Medicine, Pontificia Universidad Catolica de Chile, Santiago, Chile; 25 Red de Salud UC Christus, Santiago, Chile; 26 National Health Commission Key Laboratory of Neonatal Diseases, Children’s Hospital of Fudan University, Shanghai, China; 27 Department of Pediatrics, University of Alberta, Edmonton, Canada; 28 Fondazione IRCCS Ca’ Granda Ospedale Maggiore Policlinico, Milan, Italy; 29 Department of Clinical and Community Sciences, Department of Excellence 2023-2027, University of Milan, Milan, Italy; 30 Hospital Universitario 12 de Octubre, Madrid, Spain; 31 The National Institute for Mother and Child Care Alessandrescu-Rusescu, Polizu Maternity, Bucharest, Romania; 32 Hospital Universitario Puerta del Mar, Cadiz, Spain; 33 1st Department of Neonatology and Intensive Care Unit of Aristotle University, Ippokrateion General Hospital of Thessaloniki, Thessaloniki, Greece; 34 Department of Neonatology, Hospital Clínic Barcelona, BCNatal-Barcelona Center for Maternal-Fetal and Neonatal Medicine, Barcelona, Spain; 35 Hospital Universitario Virgen Las Nieves, Granada, Spain; 36 Department of Pediatrics II, Medical University of Innsbruck, Innsbruck, Austria; 37 Department of Neonatology and Neonatal Intensive Care Unit, University Hospital of Heraklion, Heraklion, Greece; 38 Institute of Clinical Medicine, University of Copenhagen, Copenhagen, Denmark; 39 Department of Regional Health Research, The Faculty of Health Sciences, University of Southern Denmark, Odense, Denmark

**Keywords:** Randomised clinical trial, Protocol, Neonatology, Cerebral oximetry monitoring, Mechanical ventilation

## Abstract

**Background:**

The use of invasive mechanical ventilation in newborns has several risks, including ventilator-induced lung injury, ventilation-associated pneumonia, and hyperventilation leading to potential brain injury, prolonged hospitalisations, and increased mortality. The SafeBoosC-IIIv trial aims to assess the benefits and harms of treatment guided by cerebral oximetry monitoring in newborns receiving invasive mechanical ventilation within the first 28 days from birth. The SafeBoosC consortium is a global network of neonatologists from multiple neonatal intensive care units worldwide.

**Methods:**

The SafeBoosC-IIIv trial is an investigator-initiated, multinational, randomised, pragmatic, superiority phase III clinical trial. The trial will be conducted in two steps. This is a protocol for step one. The objective of step one is to assess if treatment guided by cerebral oximetry monitoring according to the SafeBoosC treatment guideline compared with usual care in newborns receiving invasive mechanical ventilation increases the number of hospital-free days. The participants will be newborns with a gestational age more than or equal to 28 + 0 weeks, postnatal age less than 28 days, expected to receive invasive mechanical ventilation (intubation) for at least 24 h, and a cerebral oximeter available so monitoring can be started within 6 h after initiation of invasive mechanical ventilation. Exclusion criteria will be suspicion of or confirmed brain injury or congenital heart malformation likely to require surgery. A total of 1,610 participants will be randomised 1:1 to treatment guided by cerebral oximetry monitoring or usual care. The primary outcome will be hospital-free days, and the secondary outcomes will be serious adverse events and invasive mechanical ventilation-free days. All outcomes will be assessed at 90 days after randomisation.

**Discussion:**

The SafeBoosC-IIIv trial has several strengths, including detailed predefined methodology, a high degree of external validity due to centres in several countries, and the trial will be built upon the already established SafeBoosC consortium and the experience from the previous SafeBoosC-II and SafeBoosC-III trials. The SafeBoosC-IIIv trial has limitations, including current lack of funding for step two, lack of blinding of the clinical staff, and the clinicians may have limited experience in using the device.

**Trial registration:**

ClinicalTrials.gov: NCT05907317, registered 8 June 2023, https://clinicaltrials.gov/study/NCT05907317?cond=NCT05907317&rank=1

**Supplementary Information:**

The online version contains supplementary material available at 10.1186/s13063-026-09631-5.

## Background {9a-b, 10}

Newborn infants may need invasive mechanical ventilation due to various conditions [1, 2] such as respiratory distress syndrome, meconium aspiration, severe infection leading to sepsis, persistent pulmonary hypertension, congenital malformations, or acute conditions requiring surgery [3]. Specifically for preterm infants, there is a risk of respiratory failure due to underdeveloped lungs and a lack of surfactant [4]. The use of invasive mechanical ventilation comes with risks, including traumatic lung injury such as pneumothorax, ventilation-associated pneumonia, and hyperventilation, leading to compromised cerebral flow and potential brain damage [1, 2, 5]. Even at full term, newborns’ cardiorespiratory systems are not fully developed, and they have limited cardiovascular reserves, making invasive mechanical ventilation particularly complex [6].

We have limited outcome data on this diverse group of newborns receiving invasive mechanical ventilation. Within 10 neonatal intensive care units of the SafeBoosC consortium, 8.2% of the 500 newborns who required invasive mechanical ventilation died before discharge (SafeBoosC consortium, unpublished data from 2019). Danish studies revealed that 18% of children born at term who received mechanical ventilation as newborns required additional educational support, which is 2.5 times the normal rate (Wiingreen et al., unpublished data), while they also had a fourfold increased risk of cerebral palsy [7]. Thus, newborns receiving invasive mechanical ventilation represent a high-risk population with increased morbidity, prolonged hospital admissions, mortality, and risk of neurodevelopmental impairment [8].

Due to a lack of oxygen and blood flow, infants may be susceptible to organ ischemia and brain damage. Therefore, cerebral oximetry by near-infrared spectroscopy has been proposed as a method for early detection to optimise cardiovascular and respiratory support. Analysis of 23 previous trials in both children and adults suggests this approach could be beneficial, but a definitive conclusion requires more participants [9].

Given the inconclusive data from previous trials, we aim to assess whether cerebral oximetry monitoring by non-invasive near-infrared spectroscopy, along with a treatment guideline for its use, will increase hospital-free days and survival, and improve neurodevelopmental outcomes in infants receiving invasive mechanical ventilation during the neonatal period [10]. To our knowledge, no randomised clinical trials have previously evaluated treatment guided by cerebral oximetry monitoring in newborns receiving invasive mechanical ventilation.

The SafeBoosC-IIIv trial is the latest in a series of trials on treatment guided by cerebral oximetry monitoring in newborns. The SafeBoosC-II trial tested whether treatment guided by cerebral oximetry could reduce the burden of cerebral hypo- and hyperoxia during the first three days following birth in extremely preterm infants [11]. The trial showed a significant burden reduction in the treatment guided by cerebral oximetry group compared to usual care, primarily driven by a reduction in cerebral hypoxia. The subsequent SafeBoosC-III trial, a larger study of 1,601 extremely preterm infants, found that treatment guided by cerebral oximetry monitoring did not influence the risk of death or severe brain injury at 36 weeks postmenstrual age [12].

The SafeBoosC-IIIv trial will be conducted in two steps with separate objectives: In step one, we aim to assess whether treatment guided by cerebral oximetry monitoring can increase the number of hospital-free days within 90 days of randomisation in 1,610 newborns receiving invasive mechanical ventilation. Funding has been obtained for the central costs of step one. If funding is obtained for step two, we aim to assess the effect on a composite of death, neurodevelopmental disability, and non-verbal cognitive score at a 2-year follow-up. If funding is obtained for step two, we will continue to include participants until a total of 3,000 are randomised and follow all participants until 2 years of corrected age. The protocol for step two is described elsewhere [13]. The present protocol will describe step one of the trial.

## Methods

The primary hypothesis for step one is that treatment guided by cerebral oximetry monitoring according to the SafeBoosC treatment guideline will increase the number of hospital-free days within 90 days of randomisation.

### Trial design {2, 5, 12}

**Fig. 1:**
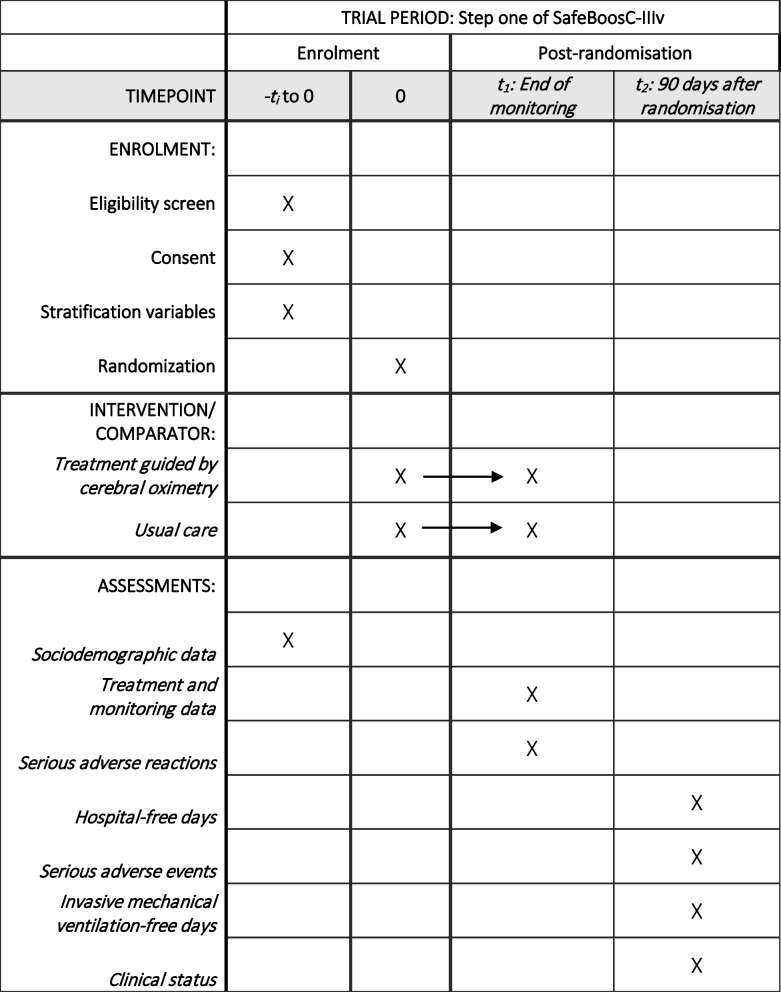
Participant timeline. Schedule of enrollment, interventions, and assessments. Citation: Chan A-W, Boutron I, Hopewell S, Moher D, Schulz KF, et al. SPIRIT 2025 statement: updated guideline for protocols of randomised trials. BMJ 2025;389:e081477. https://dx.doi.org/10.1136/bmj-2024-081477. © 2025 Chan A-W et al. This is an Open Access article distributed under the terms of the Creative Commons Attribution License (https://creativecommons.org/licenses/by/4.0/), which permits unrestricted use, distribution, and reproduction in any medium, provided the original work is properly cited

The trial is an investigator-initiated, multinational, randomised, pragmatic, superiority phase III clinical trial. Multiple neonatal intensive care units worldwide will be randomising 1,610 newborns in step one. A main publication will be published after step one has been completed. Randomisation began in April 2025. A detailed protocol was available before randomisation at www.safeboosc.eu (current version: 2.2 dated 02.06.2025). The trial protocol aligns with the SPIRIT guidelines (Supplemental Material) [14]. Please see Fig. 1 for the SPIRIT participant timeline (Fig. 1).

### Inclusion criteria {14a}

Inclusion criteria will be:

- Newborns with gestational age more than or equal to 28 + 0
- Postnatal age less than 28 days
- Expected to receive invasive mechanical ventilation (intubation) for at least 24 h, as judged by the physician intending to randomise
- Parental informed consent, unless the centre has chosen to use ‘opt-out’ or deferred consent as consent method
- A cerebral oximeter available so monitoring can be started within six hours after initiation of invasive mechanical ventilation

### Exclusion criteria {14a}

Exclusion criteria will be:

- Suspicion of or confirmed brain injury or disorder (e.g. severe hypoxic-ischemic encephalopathy, intraventricular haemorrhage grade 3 or 4, cerebral malformation, genetic or metabolic disease)
- Suspicion or diagnosis of congenital heart malformations likely to require surgery

### Participation in other trials {15d}

Participants can enrol in other trials, if such trials do not interfere with the SafeBoosC-IIIv trial design (i.e. accessing cerebral oxygenation values in the usual care group or excluding a treatment from the SafeBoosC treatment guideline) [15].

### Participant discontinuation and withdrawal {15b, 15d, 25b}

At all times, the parents may withdraw their newborns from the intervention. They may also decline the use of any future data. It will be recorded if parents provide a reason for discontinuation or withdrawal of consent. The attending physician has the authority to withdraw a participant from the intervention if safety concerns arise. There are no pre-specified criteria for withdrawal of participants from the intervention for safety concerns. Participants withdrawn from the intervention will be followed up and the participants will not be replaced.

### Recruitment {20}

On average, 1.8 newborns were randomised daily in the SafeBoosC-III trial (www.safeboosc.eu). Due to the differences in eligibility criteria from the SafeBoosC-III trial, admission records from hospitals within the SafeBoosC consortium, indicate that the number of eligible participants for SafeBoosC-IIIv may be higher [13]. Moreover, the consortium will expand to include additional countries and centres.

### Randomisation {21a-b, 22, 23}

Web-based randomisation will be centrally performed. The block sizes will vary, be computer-generated, and concealed for all investigators. Randomisation will be 1:1, stratified for centre, gestational age (above or below 34 completed weeks), and expected surgery (yes or no). On-site clinicians will use the open-source data management system OpenClinica® to screen and randomise newborns. The allocation list is generated by a data manager using randomisation generator software developed at the Copenhagen Trial Unit and uploaded to OpenClinica. The allocation list will be concealed from all staff members and clinicians except the data manager.

### Blinding {24a-c}

Due to the nature of the experimental intervention of the SafeBoosC-IIIv trial, it is difficult to blind the clinical staff and the parents. To minimise bias in assessing the primary outcome, the assessment must be performed by a person blinded to group allocation. The assessment of the primary outcome is based on a review of clinical records and contact with the parents. The principal investigator from each centre must develop a local blinding procedure, describing how blinding is achieved. Before initiating randomisation in each centre, the local blinding procedure must be approved by the trial manager on behalf of the Steering Committee. A Standard Operation Procedure with a suggested blinding method has been developed to support the principal investigators in this work.

The statisticians, trial manager, sponsor, writers of the two final abstracts before unblinding of the groups (one assuming ‘A’ to be the experimental group and one assuming ‘B’ to be the experimental group), and assessors of the primary outcome will be blinded. The independent Data Monitoring and Safety Committee (DMSC) will be provided statistics per allocation group named A and B but can request unblinding.

### Interventions {9b, 15a, 15d, 18}

Participants randomised to the experimental group will receive treatment guided by cerebral oximetry monitoring added to usual care. If possible, cerebral oximetry monitoring will be initiated before intubation or as soon as possible and within 6 h after invasive mechanical ventilation has been initiated at the latest. Treatment guided by cerebral oximetry monitoring will continue in the neonatal intensive care unit, until one of four stop criteria is met: (1) the cardiopulmonary function has been stabilised as indicated by the need for respiratory and circulatory support and evaluated by the responsible physician, (2) the infant is extubated, (3) the infant has reached 28 days after birth, or (4) the infant has deceased. During treatment guided by cerebral oximetry monitoring, the attending physician may adjust cardiorespiratory support according to the SafeBoosC treatment guideline to keep cerebral oxygenation above the predefined, sensor-specific hypoxic threshold [15]. The treatment options listed in the SafeBoosC treatment guideline are at the discretion of the treating physician and the treating team, based on a comprehensive evaluation of the newborn's clinical situation, local protocols, and guidelines. All oximeters and sensor combinations approved for clinical use in newborns and calibrated in a blood-lipid phantom to determine the hypoxic intervention threshold, corresponding to 55% oxygenation as measured with the INVOS adult sensor [16, 17], may be used. The sensor-specific thresholds are listed in the supplementary file (Supplemental Material). Randomisation will only direct the use of cerebral oximetry combined with the SafeBoosC treatment guideline during the first invasive mechanical ventilation episode. If the infant is reintubated, cerebral oximetry may be used at the discretion of the responsible physician. Also, in case of care outside the neonatal intensive care unit, e.g. during surgery, the use of cerebral oximetry is at the discretion of the responsible physicians.

Usual care will be provided in both the experimental group and control group. For newborn infants on mechanical ventilation, this often includes cardiovascular support as well as non-intensive care such as antibiotics, nutrition, etc. There will be no attempt to standardise’usual care’ across centres. Participants randomised to the control group will receive usual care without the use of cerebral oximetry monitoring during the first episode of invasive mechanical ventilation. This pragmatic approach reflects the challenges associated with controlling the use of cerebral oximetry during subsequent episodes of invasive mechanical ventilation, for example at other hospitals.

### Trial duration

The recruitment is expected to be completed within 36 months. Therefore, the primary outcome is expected to be known for the last participant 90 days later. The results based on the present protocol will be published after 1,610 participants have been followed up. If funding is obtained for step two, randomisation will continue to 3,000 participants, and the results of the 2-year follow-up will be published when all data are available [13].

### Outcomes {16–17}

#### Primary outcome

The primary outcome will be hospital-free days within 90 days of randomisation.

#### Rationale for the primary outcome

Hospital-free days, a commonly used outcome in intensive care trials [18–20], are chosen as the primary outcome for several reasons. First, hospital-free days must be considered an outcome with value to patients in themselves; they must be regarded as important to patients and their relatives to spend as little time as possible in the hospital. They may also be a valid surrogate outcome for other patient-important outcomes, as a low number of hospital-free days will likely correlate with complications and difficulties in breathing and feeding [18]. Hospital-free days measure the cumulative time in the hospital during a given observation period. If a given participant is discharged too soon, it may result in readmissions, which would also be reflected in hospital-free days [18]. Second, results based on hospital-free days reflect both incidences of deaths and the cumulated hospital burden. In contrast, the outcome length of stay is a potentially biased outcome because participants who are discharged early but frequently readmitted will contribute to a short length of stay, thereby potentially skewing the results [21]. Third, hospital-free days can be analysed as count data, often resulting in a lower sample size compared to mortality as a dichotomous outcome [18].

#### Secondary outcomes

The secondary outcomes will be participants with one or more serious adverse events and invasive mechanical ventilation-free days within 90 days of randomisation.

Serious adverse events will be defined as follows: death from any cause, bronchopulmonary dysplasia, any brain injury diagnosed by imaging (if both ultrasound and MRI are available, MRI will be prioritised), seizures treated with antiepileptic medicine, haemodynamic insufficiency that needs cardiovascular support, spontaneous bowel perforation or necrotising enterocolitis defined as Bell’s grade 2 or more [22], extracorporeal membrane oxygenation treatment, renal replacement therapy, or nosocomial infection. The investigators reviewing the clinical records will decide and diagnose if a serious adverse event has occurred at follow-up. Recommended definitions may be found in the supplementary file (Supplemental Material).

#### Exploratory outcomes

The exploratory outcomes will be late-onset sepsis and invasive mechanical ventilation-related infection within 90 days of randomisation.

### Statistical plan and data analysis {5, 27a-d}

A comprehensive statistical analysis plan has be developed and published prior to the unblinding of data [23]. General principles are outlined below.

All analyses will be conducted according to the intention-to-treat principle, i.e. all randomised participants with available data will be included in the groups to which they were randomised in all analyses [23].

We will assess whether the statistical and clinical significance thresholds are crossed (Bayes factor calculations will be reported in supplementary material) [24]. Our primary conclusion will be based on the primary outcome, so all tests of statistical significance (including subgroup analyses) will be two-sided with a threshold of 5% [24]. *P* values will only be reported for the primary outcome [23].

Van Elteren test stratified by centre will be used to analyse the two count data outcomes (hospital-free days and invasive mechanical ventilation-free days within 90 days of randomisation) and Hodghes-Lehmann median differences, and 95% Hodghes-Lehmann confidence intervals will be reported. Mixed-effects logistic regression with stratification variables as fixed effect and centre as random effects will be used to analyse the dichotomous outcomes.

The primary analysis will include all randomised participants with available data. Missing data will be handled according to the recommendations by Jakobsen et al. [25]. In short, missing data will be considered negligible if the amount of missing data is less than 5%. If the amount of missing data is more than 5%, we will apply multiple imputation and best–worst/worst–best case scenario analyses to evaluate the potential impact of missing data on the results, depending on the amount of missing data [23, 25].

We will perform the following subgroup analyses:

- Sex (male or female)
- Indication for invasive mechanical ventilation (respiratory insufficiency or surgery)
- Race or ethnic group (White, Black or African American, Asian, Hispanic or Latino, Mixed, or other)

Subgroup analyses will primarily be presented in forest plots. Additionally, subgroup comparisons will be conducted using tests of interaction. Subgroup analyses will be hypothesis-generating rather than confirmatory [23].

### Sample size {19}

The primary outcome of hospital-free days is estimated using the Danish national registry. Between 2001 and 2018, 1,392 infants born at gestational age more than or equal to 28 weeks received invasive mechanical ventilation, had an Apgar score of > 6 at 5 min, and did not have any heart defects. Of those, 140 (10.1%) died within 90 days, and the median length of stay of the survivors was 26 days. We ran simulations with 1,000 iterations for each sample size to estimate the sample size. Based on a relative risk reduction of death of 5% and an absolute increase of 3 days in the estimate of hospital-free days of the surviving participants (considered clinically relevant) with an alpha of 0.05 and a power of 90% using the van Elteren test, we would need to include 1,610 participants (Supplemental Material).

### Power of the secondary outcomes

Based on data from Statistics Denmark and Rigshospitalet [26, 27], we assume 20% of the newborns receiving invasive mechanical ventilation will experience one or more serious adverse events within 90 days of randomisation. With a relative risk reduction of 30% in the experimental group, we can detect this difference between the experimental and control groups with 89% power at a 5% significance level.

For invasive mechanical ventilation-free days, we defined a clinically relevant relative risk reduction of 5% and an absolute increase of 3 days in the estimate. Based on an alpha of 0.05 using the van Elteren test, we would have a power of 100% when including 1,610 participants (Supplemental Material).

### Power of the exploratory outcomes

Based on data from Statistics Denmark and Rigshospitalet [26, 27], we anticipate that 14.6% of the newborns receiving invasive mechanical ventilation will experience late-onset sepsis within 90 days of randomisation. With a relative risk reduction of 20% in the experimental group, we will be able to detect this difference between the experimental and the control group with 41% power at a 5% significance level. Due to the limited statistical power, this outcome will be assessed as exploratory only.

Based on data from Statistics Denmark and Rigshospitalet [26, 27], we anticipate that 14% of the newborns receiving invasive mechanical ventilation will experience invasive mechanical ventilation-related infection within 90 days of randomisation. With a relative risk reduction of 20% in the experimental group, we can detect this difference between the experimental and the control group with 40% power at a 5% significance level. Due to the limited statistical power, this outcome will be assessed as exploratory only.

### Organisation {3c-d, 13}

The trial organisation consists of the Steering Committee, Executive Steering Committee, principal investigators from all centres, and the Copenhagen Trial Unit management group including the sponsor and trial managers. The Steering Committee includes a national coordinator from each country as well as the trial managers and sponsor. The Steering Committee serves as the decision-making body for the trial. The Executive Steering Committee is a smaller group of neonatologists that supports the trial managers and sponsor with day-to-day management. The sponsor and trial managers are responsible for daily management and communication with the principal investigators. With the support of the Steering Committee and Executive Steering Committee, the sponsor and trial managers have provided the trial infrastructure, including English versions of the protocol, templates, standard operating procedures for trial procedures, consent forms, participant information sheets, the electronic case report form (eCRF), website, and web-based training material.

The trial organisation currently includes active centres and centres in the preparation phase in the following countries: Argentina, Australia, Austra, Belgium, Brazil, Canada, Chile, China, the Czech Republic, Denmark, Germany, Greece, India, Ireland, Italy, Poland, Romania, Spain, Switzerland, Türkiye, the United States, and Zambia. Please find the updated list of centres at www.safeboosc.eu.

### Centre and staff eligibility {14b}

Neonatal intensive care units are eligible if they have implemented, or will implement, treatment guided by cerebral oximetry with a high level of clinical competence. The responsible physician and clinical staff must have equipoise regarding the clinical value of treatment guided by cerebral oximetry in newborns receiving invasive mechanical ventilation. Furthermore, each centre must expect to randomise at least 10 participants per year.

### Training of clinical staff {14b, 15c, 25a}

Clinical staff at all centres will be offered web-based training [28] before caring for trial participants. The training includes modules on the protocol, cerebral oximetry monitoring, local monitoring, and the SafeBoosC treatment guideline. However, it will pragmatically be up to the responsible clinician to use cerebral oximetry based on the SafeBoosC treatment guideline [15].

### Safety {28a}

During the trial, mortality and neonatal morbidities at 90 days of life, including serious adverse reactions and serious adverse events will be monitored by the DMSC. Serious adverse reactions include (1) physical mishaps associated with managing the oximeter and sensors, and (2) clinical mismanagement based on data from the cerebral oxygenation monitoring.

The charter for the DMSC is available in the supplementary file (Supplemental Material) and at www.safeboosc.eu.

### Data management {25a, 26, 33}

The Copenhagen Trial Unit will provide central, web-based data entry in the eCRF. As in the SafeBoosC-II and SafeBoosC-III trials, OpenClinica® will be used for data management [11, 12]. On-site clinicians will report data in the eCRF using standardised forms (available at www.safeboosc.eu) that have been developed by the Copenhagen Trial Unit management group and tested by members of the Executive Steering Committee. Separate forms will be used for each time point. Clinicians will not be able to submit forms unless all required data are reported or the trial manager has been informed of the reasons for any missing data. Protocol deviations will be reported in the eCRF.

### Monitoring {3d, 15c, 29}

The trial manager, the coordinating investigator, the Copenhagen Trial Unit, and members of the Executive Steering Committee will perform thorough and systematic central data monitoring. Central checks will be performed monthly with focus on recruitment to the trial, quality, completeness, and timeliness of data entry in the eCRF. Olsen et al. has described the principles of the central data monitoring elsewhere [29]. The specific SafeBoosC-IIIv trial central data monitoring plan is available in the supplementary file (Supplemental Material) and at www.safeboosc.eu.

Local data monitoring will be performed in accordance with the International Conference on Harmonization’s Good Clinical Practice guideline [30]. The following will be locally monitored: the existence of a clinical file for all participants, the presence of documented informed consent, and the entry of trial participation in clinical files. The local investigator will enter data for group allocation, gestational age, as well as survival status and hospital-free days at 90 days after randomisation based on data reported by the blinded outcome assessor. An independent monitor will conduct source checks. If required by local regulations for participating centres, additional data monitoring may be conducted.

### Ethical considerations {30, 32a}

Due to the pathophysiology of newborns, the question on potential benefits and harms of treatment guided by cerebral oximetry monitoring in newborns receiving invasive mechanical ventilation newborns can only be answered by a trial in this particular population. Clinical practice regarding the use of cerebral oximetry in newborns varies considerably between hospitals and countries, with uncertainty regarding the potential beneficial or harmful effects. The use of cerebral oximetry carries a risk of skin injury and incurs increased costs in terms of staff time and equipment expenses. The neutral results of the SafeBoosC-III trial may support the uncertainty of benefits or harms. The disadvantage in terms of risk of skin injury and disturbance of the patient may be less in larger, more mature infants, who are already under continuous monitoring by multiple medical devices.

The protocol for SafeBoosC-IIIv has been approved by the Danish Research Ethics Committees (Ethics committee number: 2402572, all versions available at www.safeboosc.eu). Before randomisation, all participating neonatal intensive care units must have their eligibility confirmed and have the protocol approved by the relevant ethics committee. Cerebral oximetry is currently used in many neonatal intensive care units worldwide, and all interventions proposed in the SafeBoosC treatment guideline are well-known for treating this patient group. Therefore, if approved by the ethics committee, the methods used to obtain informed consent from parents may be decided upon by the national or local investigator as prior, opt-out, deferred, or a combination of these methods.

### Protocol amendments {31}

The sponsor and trial manager will be responsible for any amendments to the protocol. If relevant, the funder will be notified. The updated protocol will be stored in the sponsor’s trial master file. In addition, the trial manager will share the updated protocol with all principal investigators for addition to the local investigator site files. The protocol will also be updated on ClinicalTrials.gov.

### Publication plan and data sharing {4–6, 8, 11}

The trial protocol has been registered on ClinicalTrials.gov (NCT05907317, first submitted on 8 June 2023). All positive, neutral, and negative results will be published in peer-reviewed international journals, if possible. If data are not published, summary data of entry data, stratification variables, randomisation, and outcomes will be uploaded. An anonymised, recoded dataset will be made available for research, as approved by the trial Steering Committee when the main publication and substudies are published to minimise the risk of re-identification of participants. Patients are not involved in the design, conduct, or reporting of this trial.

## Discussion

The SafeBoosC-IIIv trial is an investigator-initiated, multinational, randomised, pragmatic, superiority phase III clinical trial. The objective of the SafeBoosC-IIIv trial is to evaluate the benefits and harms of treatment guided by cerebral oximetry monitoring compared with usual care in newborns receiving invasive mechanical ventilation. Participants will be randomly assigned from multiple neonatal intensive care units worldwide. The trial will be conducted in two steps, with the current protocol focusing on step one. The primary outcome for step one will be hospital-free days, and the secondary outcomes will be serious adverse events and invasive mechanical ventilation-free days, all assessed at 90 days after randomisation.

The SafeBoosC-IIIv trial has several strengths. First, the methodology is described in detail and is predefined, and the protocol is reported according to the SPIRIT guidelines. Furthermore, a statistical analysis plan detailing the analyses will be published before unblinding the data to prevent data-driven results. Second, the SafeBoosC-IIIv trial will have a high degree of external validity due to the inclusion of participants from several countries and the limited number of exclusion criteria. Third, the SafeBoosC-IIIv trial will build upon the already established SafeBoosC consortium and the experience from the previous SafeBoosC-II and SafeBoosC-III trials. Fourth, the SafeBoosC-IIIv trial is a pragmatic trial with few outcomes and follow-up time points, which makes it possible to run such a large trial across several countries and continents.

The SafeBoosC-IIIv trial also has limitations. First, it is a considerable limitation that step two of the present trial has yet to be funded. Therefore, it is currently uncertain whether step two will be conducted. Although the primary outcome of hospital-free days has clinical relevance, the outcomes for step two may be considered more patient-important. Second, the clinical staff involved in the immediate care of the newborns cannot be blinded to the allocated intervention. Third, some clinicians may have limited experience in using the device. Web-based training will be offered to all clinicians to accommodate this challenge. The training materials are available for download, enabling the principal investigator to provide alternative options**,** e.g. group sessions**,** when individual web-based training is not feasible. Fourth, we did not systematically involve patients or patient unions in constructing the trial and trial protocol.

For more than a decade, the SafeBoosC consortium has conducted trials on treatment guided by cerebral oximetry monitoring in newborns. While SafeBoosC-IIIv was built on the foundation of previous SafeBoosC trials, the infrastructure and organisation were rebuilt for each trial. These are costly and time-consuming aspects of clinical trials, which delay the conduct of new trials. In recent years, the platform trial design has gained interest within different medical specialities, including neonatology [31]. While the traditional randomised clinical trial typically assesses one intervention before stopping the trial, the platform trial reuses the same platform to assess several interventions without stopping the trial [32, 33]. This allows a platform trial to maintain an established infrastructure, and fewer resources are wasted by building long-term platforms to assess several interventions [34, 35]. New interventions may be added in parallel or sequentially, and once the sample size for an arm is reached, the comparison can be removed from the platform, analysed, and published [36]. Participants may be included in multiple comparisons, e.g. as part of a shared control arm, which decreases the need for participants and, thereby also, time and costs [35]. Accordingly, the SafeBoosC consortium, with its extensive, international collaboration and experience in building trial infrastructures for large-scale pragmatic trials, provides an optimal opportunity to launch a platform trial for a more efficient, cost-effective, and timesaving approach to improving outcomes for newborns.

### Trial status

All versions of the protocol are available at www.safeboosc.eu, with the current version being 2.2 dated 2 June 2025. Recruitment began on 9 April 2025, with the first participant being randomised on 11 April 2025. Randomisation is expected to be completed within 29 months, i.e. before September 2027. More than 70 neonatal intensive care units across six continents have currently initiated trial preparations.

## Supplementary Information

Aditional file 1: Treatment guided by cerebral oximetry in newborns receiving invasive mechanical ventilation: study protocol for step one of the SafeBoosC-IIIv randomised clinical trial.

## Data Availability

Not applicable.

## Abbreviations

DMSC: Data Monitoring and Safety Committee
eCRF: Electronic case report form
SafeBoosC: Safeguarding the Brain of our smallest Children
