# Supplementary material for: Treatment guided by cerebral oximetry in newborns receiving invasive mechanical ventilation: study protocol for step one of the SafeBoosC-IIIv randomised clinical trial
Source: Trials. 2026 Mar 17;27:318. doi: 10.1186/s13063-026-09631-5 (PMC13107782; doi:10.1186/s13063-026-09631-5)
Supplement: Supplementary file 1 — Aditional file 1: Treatment guided by cerebral oximetry in newborns receiving invasive mechanical ventilation: study protocol for step one of the SafeBoosC-IIIv randomised clinical trial. [file 13063_2026_9631_MOESM1_ESM.docx]

**Supplementary material for:**

**Treatment guided by cerebral oximetry in newborns receiving invasive mechanical ventilation: study protocol for step one of the SafeBoosC-IIIv randomised clinical trial**

For updated information and the full protocol, please see [www.safeboosc.eu](http://www.safeboosc.eu)

**​**

Spons​​or information {3b}

Sponsor: Janus Christian Jakobsen

Mail: janus.jakobsen@ctu.dk

Trial Manager: Caroline Barkholt Kamp

Mail: caroline.kamp@ctu.dk

Copenhagen Trial Unit, Centre for Clinical Intervention Research, The Capital Region of Denmark, Copenhagen University Hospital – Rigshospitalet, Copenhagen, Denmark

Table of contents

[SPIRIT 2025 checklist 3](#_Toc222492163)

[Sensor-specific thresholds 8](#_Toc222492164)

[Definitions of serious adverse events 9](#_Toc222492167)

[Sample size {19} 11](#_Toc222492168)

[Charter for the independent Data Monitoring and Safety Committee {3d, 28a-b, 29} 17](#_Toc222492169)

[World Health Organization Trial Registration Data Set (Version 1.3.1) {1b} 41](#_Toc222492191)

## SPIRIT 2025 checklist

| **Section / Topic** | **No** | **SPIRIT 2025 checklist item description** | **Reported in section:** |
| --- | --- | --- | --- |
| **Administrative information** | | |  |
| Title and structured summary | 1a | Title stating the trial design, population, and interventions, with identification as a protocol | Front page, p.1 |
|  | 1b | Structured summary of trial design and methods, including items from the World Health Organization Trial Registration Data Set | Supplementary material, p.41 |
| Protocol version | 2 | Version date and identifier | Trial design, p.8 |
| Roles and responsibilities | 3a | Names, affiliations, and roles of protocol contributors | Front page, p.1, Authors’ contributions, p.21 |
|  | 3b | Name and contact information for the trial sponsor | Supplementary material, p.1 |
|  | 3c | Role of trial sponsor and funders in design, conduct, analysis, and reporting of trial; including any authority over these activities | Organisation, p.14,  Funding statement, p.20 |
|  | 3d | Composition, roles, and responsibilities of the coordinating site, steering committee, endpoint adjudication committee, data management team, and other individuals or groups overseeing the trial, if applicable | Organisation, p.14,  Monitoring, p.16, Supplementary material, p.17 |
| **Open science** | | |  |
| Trial registration | 4 | Name of trial registry, identifying number (with URL), and date of registration. If not yet registered, name of intended registry | Abstract, p.5, Publication plan and data sharing, p.17 |
| Protocol and statistical analysis plan | 5 | Where the trial protocol and statistical analysis plan can be accessed | Abstract, p.5, Trial design, p.7, Statistical plan and data analysis, p.12, Publication plan and data sharing, p.17 |
| Data sharing | 6 | Where and how the individual de-identified participant data (including data dictionary), statistical code, and any other materials will be accessible | Publication plan and data sharing, p.17 |
| Funding and conflicts of interest | 7a | Sources of funding and other support (e.g., supply of drugs) | Funding statement, p.20 |
|  | 7b | Financial and other conflicts of interest for principal investigators and steering committee members | Conflicts of interest statement, p.20 |
| Dissemination policy | 8 | Plans to communicate trial results to participants, healthcare professionals, the public, and other relevant groups (e.g., reporting in trial registry, plain language summary, publication) | Publication plan and data sharing, p.17 |
| **Introduction** | | |  |
| Background and rationale | 9a | Scientific background and rationale, including summary of relevant studies (published and unpublished) examining benefits and harms for each intervention | Background, p.6 |
|  | 9b | Explanation for choice of comparator | Background, p.6,  Interventions, p.10 |
| Objectives | 10 | Specific objectives related to benefits and harms | Background, p.6 |
| **Methods: Patient and public involvement, trial design** | | |  |
| Patient and public involvement | 11 | Details of, or plans for, patient or public involvement in the design, conduct, and reporting of the trial | Publication plan and data sharing, p.17 |
| Trial design | 12 | Description of trial design including type of trial (e.g., parallel group, crossover), allocation ratio, and framework (e.g., superiority, equivalence, non-inferiority, exploratory) | Trial design, p.7 |
| **Methods: Participants, interventions, and outcomes** | | |  |
| Trial setting | 13 | Settings (e.g., community, hospital) and locations (e.g., countries, sites) where the trial will be conducted | Organisation, p.14 |
| Eligibility criteria | 14a | Eligibility criteria for participants | Inclusion criteria, p.8, Exclusion criteria, p.8 |
|  | 14b | If applicable, eligibility criteria for sites and for individuals who will deliver the interventions (e.g., surgeons, physiotherapists) | Centre and staff eligibility, p.15, Training of clinical staff, p.15 |
| Intervention and comparator | 15a | Intervention and comparator with sufficient details to allow replication including how, when, and by whom they will be administered. If relevant, where additional materials describing the intervention and comparator (e.g., intervention manual) can be accessed | Interventions, p.10 |
|  | 15b | Criteria for discontinuing or modifying allocated intervention/comparator for a trial participant (e.g., drug dose change in response to harms, participant request, or improving/worsening disease) | Participant discontinuation and withdrawal, p.8 |
|  | 15c | Strategies to improve adherence to intervention/comparator protocols, if applicable, and any procedures for monitoring adherence (e.g., drug tablet return, sessions attended) | Training of clinical staff, p.15, Monitoring, p.16 |
|  | 15d | Concomitant care that is permitted or prohibited during the trial | Participation in other trials, p.8, Participant discontinuation and withdrawal, p.8, Interventions, p.10 |
| Outcomes | 16 | Primary and secondary outcomes, including the specific measurement variable (e.g., systolic blood pressure), analysis metric (e.g., change from baseline, final value, time to event), method of aggregation (e.g., median, proportion), and time point for each outcome | Outcomes, p.11 |
| Harms | 17 | How harms are defined and will be assessed (e.g., systematically, non-systematically) | Outcomes, p.11 |
| Participant timeline | 18 | Time schedule of enrollment, interventions (including any run-ins and washouts), assessments, and visits for participants. A schematic diagram is highly recommended (see Figure) | Interventions, p.10, Figure 1 |
| Sample size | 19 | How sample size was determined, including all assumptions supporting the sample size calculation | Sample size, p.13, Supplementary material, p.11 |
| Recruitment | 20 | Strategies for achieving adequate participant enrollment to reach target sample size | Recruitment, p.9 |
| **Methods: Assignment of interventions** | | |  |
| Randomization: |  |  |  |
| Sequence generation | 21a | Who will generate the random allocation sequence and the method used | Randomisation, p.9 |
|  | 21b | Type of randomization (simple or restricted) and details of any factors for stratification. To reduce predictability of a random sequence, other details of any planned restriction (e.g., blocking) should be provided in a separate document that is unavailable to those who enroll participants or assign interventions | Randomisation, p.9 |
| Allocation concealment  mechanism | 22 | Mechanism used to implement the random allocation sequence (e.g., central computer/telephone; sequentially numbered, opaque, sealed containers), describing any steps to conceal the sequence until interventions are assigned | Randomisation, p.9 |
| Implementation | 23 | Whether the personnel who will enroll and those who will assign participants to the interventions will have access to the random allocation sequence | Randomisation, p.9 |
| Blinding | 24a | Who will be blinded after assignment to interventions (e.g., participants, care providers, outcome assessors, data analysts) | Blinding, p.9 |
|  | 24b | If blinded, how blinding will be achieved and description of the similarity of interventions | Blinding, p.9 |
|  | 24c | If blinded, circumstances under which unblinding is permissible, and procedure for revealing a participant’s allocated intervention during the trial | Blinding, p.9 |
| **Methods: Data collection, management, and analysis** | | |  |
| Data collection methods | 25a | Plans for assessment and collection of trial data, including any related processes to promote data quality (e.g., duplicate measurements, training of assessors) and a description of trial instruments (e.g., questionnaires, laboratory tests) along with their reliability and validity, if known. Reference to where data collection forms can be accessed, if not in the protocol | Training of clinical staff, p.15, Data management, p.16 |
|  | 25b | Plans to promote participant retention and complete follow-up, including list of any outcome data to be collected for participants who discontinue or deviate from intervention protocols | Participant discontinuation and withdrawal, p.8 |
| Data management | 26 | Plans for data entry, coding, security, and storage, including any related processes to promote data quality (e.g., double data entry; range checks for data values). Reference to where details of data management procedures can be accessed, if not in the protocol | Data management, p.16 |
| Statistical methods | 27a | Statistical methods used to compare groups for primary and secondary outcomes, including harms | Statistical plan and data analysis, p.12 |
|  | 27b | Definition of who will be included in each analysis (e.g., all randomized participants), and in which group | Statistical plan and data analysis, p.12 |
|  | 27c | How missing data will be handled in the analysis | Statistical plan and data analysis, p.12 |
|  | 27d | Methods for any additional analyses (e.g., subgroup and sensitivity analyses) | Statistical plan and data analysis, p.12 |
| **Methods: Monitoring** | | |  |
| Data monitoring committee | 28a | Composition of data monitoring committee (DMC); summary of its role and reporting structure; statement of whether it is independent from the sponsor and funder; conflicts of interest and reference to where further details about its charter can be found, if not in the protocol. Alternatively, an explanation of why a DMC is not needed | Supplementary material, p.17 |
|  | 28b | Explanation of any interim analyses and stopping guidelines, including who will have access to these interim results and make the final decision to terminate the trial | Supplementary material, p.17 |
| Trial monitoring | 29 | Frequency and procedures for monitoring trial conduct. If there is no monitoring, give explanation | Monitoring, p.16 |
| **Ethics** | | |  |
| Research ethics approval | 30 | Plans for seeking research ethics committee/institutional review board approval | Ethical considerations, p.16 |
| Protocol amendments | 31 | Plans for communicating important protocol modifications to relevant parties | Protocol amendments, p.17 |
| Consent or assent | 32a | Who will obtain informed consent or assent from potential trial participants or authorized proxies, and how | Ethical considerations, p.16 |
|  | 32b | Additional consent provisions for collection and use of participant data and biological specimens in ancillary studies, if applicable | N/A, no biological specimens were collected as part of this trial |
| Confidentiality | 33 | How personal information about potential and enrolled participants will be collected, shared, and maintained in order to protect confidentiality before, during, and after the trial | Data management, p.16 |
| Ancillary and post-trial care | 34 | Provisions, if any, for ancillary and post-trial care, and for compensation to those who suffer harm from trial participation | N/A, no ancillary/post-trial care |

*We strongly recommend reading this checklist in conjunction with the SPIRIT 2025 Explanation and Elaboration and the SPIRIT 2025 Expanded Checklist for important clarifications on all the items. We also recommend reading relevant SPIRIT extensions. See [www.consort-spirit.org](http://www.consort-spirit.org)

Citation: Chan A-W, Boutron I, Hopewell S, Moher D, Schulz KF, et al. SPIRIT 2025 statement: updated guideline for protocols of randomised trials. BMJ 2025;389:e081477. <https://dx.doi.org/10.1136/bmj-2024-081477> © 2025 Chan A-W et al. This is an Open Access article distributed under the terms of the Creative Commons Attribution License (<https://creativecommons.org/licenses/by/4.0/>), which permits unrestricted use, distribution, and reproduction in any medium, provided the original work is properly cited.

## Sensor-specific thresholds

# **Requirements for cerebral oximeters in SafeBoosC-IIIv** In general, all commercially available cerebral oximeters that are approved for clinical use in newborns (FDA-approved, CE-marked etc.) may be used in SafeBoosC-IIIv. The aim is to use multiple different devices to generate generic results. There are now seven commercially available devices in use (INVOS, NIRO, Fore-Sight, Sensmart, O3,Egos, and Oxyprem) with a total of 14 adjacent NIRS sensors. The appropriate intervention threshold, defined as the rStO2 value that reflects the same level of brain tissue oxygenation as an rStO2 value of 55% using the INVOS adult sensor, has been defined for these devices and their sensors. For any new device and sensor, the appropriate threshold will be determined.

# **Hypoxic intervention thresholds in different oximeters**

Hypoxic thresholds corresponding to a 55% rStO2 in INVOS adult. The different subtypes within each NIRS device (e.g. FORESIGHT small/FORESIGHT small band) represent different sensors and therefore show different hypoxic thresholds.

| **NIRS Device** | **Hypoxic threshold %** |
| --- | --- |
| FORESIGHT small | 66 |
| FORESIGHT non-adhesive small | 67 |
| NIRO small | 61 |
| NIRO small re-usable | 63 |
| NIRO large | 62 |
| NIRO large re-usable | 62 |
| INVOS neo | 63 |
| SenSmart neo 8004CB-NA | 66 |
| Oxyprem 1.4 re-usable | 48 |
| NOAH (OxyPrem) | Will be updated |
| O3 Pediatric | 64 |
| O3 Neonatal | 64 |
| Egos reusable pediatric/neonate | 56 |
| Egos disposable pediatric/neonate | 56 |

## Definitions of serious adverse events

*One or more serious adverse events within 90 days of randomisation: death from any cause, bronchopulmonary dysplasia,* *any brain injury diagnosed by imaging (if both ultrasound and MRI are available, MRI will be prioritized), seizures treated with antiepileptic medicine, hemodynamic insufficiency that needs cardiovascular support, spontaneous bowel perforation or necrotizing enterocolitis defined as Bell’s grade 2 or more, extracorporeal membrane oxygenation treatment, renal replacement therapy, and nosocomial infection.*

The diagnoses of any of the serious adverse events will be made by the outcome assessor using clinical records, however, the following definitions are recommended.

The following **neonatal outcomes** expressed as the **grades outlined below or above**

(adapted from: **E**uropean **N**ewborn **S**tudy: **E**arly **M**arkers for a **B**etter **L**ife-ENSEMBLE;

International Neonatal Consortium (doi: 10.1136/archdischild-2019-317399); European Standards of Care for Newborn Care (EFCNI))**.**

**Bronchopulmonary dysplasia (grade 2):**

1. Supplemental oxygen at 28 days AND need for 22-30% oxygen at 36 weeks PMA in infants born <32 weeks' gestation; need for 22-30% oxygen by 56 days postnatal age in infants born >32 weeks' gestation; need for 22-30% oxygen at discharge. Note: For conversion of oxygen administered by different modalities to FiO2, see http://nicutools.org/MediCalcs/ActualO2.php3.

**Brain injury documented either by cranial ultrasound (CU), magnetic resonance imaging (MRI), or computed tomography (CT):**

1. Peri-intraventricular hemorrhage (grade 2 or more): Hemorrhage occupying less than 50% of the ventricle volume.
2. Periventricular haemorrhagic (venous) infarction.
3. Post-haemorrhagic ventricular dilatation: ventricular dilatation with ventricular index > 4mm above the 97th percentile or anterior horn with > 6 mm or need of CSF removal (drain, shunt, reservoir, lumbal puncture).
4. Periventricular leukomalacia (grade 2) (CUs diagnosis): Transient periventricular echodensities evolving into small, localized frontal-parietal cysts or persistent diffuse echodensities.
5. Extensive punctate white matter injury (MRI diagnosis).
6. Perinatal ischemic stroke.
7. Acute HI brain damage:
   1. (CU diagnosis): i) brain swelling; ii) bilateral-symmetric basal ganglia-thalamic injury or focal (asymmetric) hyperechoic areas; iii) laminar cortical necrosis; iv) periventricular and/or subcortical white matter injury
   2. (MRI diagnosis): i) Central and/or peri-rolandic gray matter damage; ii) Hypoxic-ischemic injury in watershead areas.
8. Cerebellar injury (ischemic-haemorrhagic) involving vermis or >1/3 of hemisphere.
9. Big extra-axial bleeding or parenchymal bleeding/contusion with/without midline shift.
10. Other: imaging findings related to CNS acquired infection.

**Neonatal seizures that need for treatment**

1. Neonatal epileptic seizure (grade 2): electroencephalogram (EEG)-proven seizures that are controlled with one anti-seizure drug (no recurrence within three days after treatment).
2. Neonatal Convulsions (grade 3): Suspected seizures (no EEG) uncontrolled with one anti-seizure drug (recurrence within three days after treatment or requiring two or more anti-seizure drugs).

**Hemodynamic insufficiency**

1. Persistent hypotension (grade 3): hypotension affecting perfusion; requiring major care change (e.g., vaso-active drugs or hydrocortisone).
2. Clinical signs of compromised perfusion regardless blood pressure (raised serum lactate, poor urine output, central-to-peripheral temperature gap, prolonged skin refill-time, echocardiographic parameters) requiring major care change (e.g., vaso-active drugs or hydrocortisone).

**Bowel AE**

1. Neonatal spontaneous intestinal perforation (grade 3): presence of spontaneous intestinal perforation (SIP), non-urgent medical stabilization and surgical intervention indicated.
2. NEC (Bell’s stage 2): confirmed NEC with lack of bowel sounds, pain, and tenderness in the abdomen, low or no intestinal movement, presence of gasfilled spaces in the intestinal walls, including stage 1 clinical signs (lethargy, vomiting, bloody stools abdominal bloating, slow heart rate and unstable temperature).

**Renal replacement therapy**

1. Infants receiving any of the following therapies: peritoneal dialysis, hemodialysis, or hemofiltration.

**Nosocomial infection**

1. Late-onset sepsis: Defined as antibiotics prescribed after 72 hours and given for 5 days or more.
2. Invasive mechanical ventilation (endotracheal or tracheostomy)-related infection

## Sample size {19}

## Charter for the independent Data Monitoring and Safety Committee {3d, 28a-b, 29}

**Introduction**

This Charter defines the primary responsibilities of the independent Data Monitoring and Safety Committee (DMSC) of the pragmatic randomised clinical trial SafeBoosC-IIIv, its relationship with other trial components, its membership, and the purpose and timing of its meetings, all based on the good clinical practice (GCP) guidelines. The SafeBoosC-IIIv trial protocol is found on the trial web page ([www.safeboosc.eu](http://www.safeboosc.eu)), and it includes details on the statistics and the trial sample size calculation. A fully detailed statistical analysis plan will be developed and published before commencement of any data analyses, including the first interim analysis. This Charter also provides the procedures for ensuring confidentiality and proper communication, outlines the content of the open and closed reports, and provides a proposal for the statistical monitoring guidelines to be implemented by the DMSC.

**Primary responsibilities of the DMSC**

The DMSC will be responsible for safeguarding the interests of the trial participants as well as potential future trial participants by assessing the benefits and harms of the intervention during the trial. The DMSC will provide recommendations about stopping or continuing the trial to the Steering Committee of SafeBoosC-IIIv. A central responsibility of the DMSC is to contribute to enhancing the integrity of the trial. Results from other clinical trials or investigational plans in the same field can be weighted into the DMSCs recommendations, but any external information shall be assessed with caution.

The DMSC will be advisory to the Steering Committee. The Steering Committee will be responsible for promptly reviewing the DMSC recommendations, deciding whether to continue or terminate the trial and determining whether amendments to the protocol or changes in trial conduct are required. The DMSC is required to keep all information and data obtained from the coordinating trial unit confidential throughout the entire process, as well as keeping any results from the safety monitoring confidential.

One interim analysis is pre-planned and shall take place after one-third of the trial participants (n=536) reached follow-up of the primary outcome. The timing and prevalence of additional interim analyses will be decided solely by the DMSC. The DMSC will communicate via e-mail, telephone conference, or online meetings to evaluate the analysis of the SafeBoosC-IIIv trial. The DMSC may additionally meet or communicate, whenever they decide, to discuss the safety of the trial participants.

The sponsor has the responsibility to report to the DMSC data on the primary outcome (hospital-free days within 90 days from randomisation) and serious adverse events (SAEs) per intervention group under code for all participants, as well as serious adverse reactions (SARs) for participants in the experimental group. The DMSC can request reporting of these outcomes at any time during the trial. The DMSC will be notified of all SARs on a continuous basis, no later than a week from the initial reporting.

The recommendations of the DMSC regarding continuing, changing the design, or stopping the trial should be communicated without delay to the Steering Committee of the SafeBoosC-IIIv trial. After receiving the recommendations, the Steering Committee is responsible for informing all principal investigators as fast as possible, and no later than five working days, of the recommendation from the DMSC and the Steering Committee decision hereof.

**Members of the DMSC**

The DMSC is an independent multidisciplinary group consisting of two clinicians and a biostatistician that collectively have experience in the management of critically ill newborns and the conduct, monitoring, and analysis of randomised clinical trials, including serving as DMSC members in the SafeBoosC-III trial.

*DMSC members*

Andrew Whitelaw

Emeritus Professor of Neonatal Medicine, University of Bristol, Neonatal Neuroscience level D, St Michael’s Hospital, Bristol, United Kingdom

James Boardman

Professor of Neonatal Medicine, Centre for Clinical Brain Sciences, Centre for Reproductive Health, Institute for Regelation and Repair, Edinburgh, United Kingdom

Theis Lange

Professor, Section of Biostatistics, Department of Public Health, University of Copenhagen, Copenhagen, Denmark

**Conflicts of interest**

Such conflicts may be financial, scientific, or regulatory in nature. The DMSC membership has been restricted to individuals free of conflicts of interest.

Any DMSC members who develop significant conflicts of interest during the trial, should resign from the DMSC.

DMSC membership is to be for the duration of the clinical trial. If any members leave the DMSC during the trial, the Steering Committee shall appoint the replacement(s) in agreement with the remaining members of the DMSC.

**Proper communication**

To enhance the integrity and credibility of the trial, procedures will be implemented to ensure the DMSC has sole access to evolving information from the clinical trial, regarding comparative results of the primary outcome and safety data, aggregated under code by treatment group (0,1). An exemption will be made to permit access to the data manager, who will serve as a liaison between the database and the DMSC.

At the same time, procedures will be implemented to ensure that proper communication is achieved between the DMSC and the Steering Committee, sponsor, and investigators. An open report and a closed report format will be implemented to provide a forum for the exchange of information among the various parties who share responsibility for the successful conduct of the trial. The intent of this format is to enable the DMSC to preserve the confidentiality of the comparative safety results, while at the same time providing opportunities for interaction between the DMSC and others who have valuable insights into trial-related issues.

*Closed session*

Sessions involving only DMSC members will be held to allow discussion of confidential data from the clinical trial, including information about the benefits and harms of the intervention. In order to ensure that the DMSC will be fully informed in its primary mission of safeguarding the interest of the participants and potential future participants, the DMSC will be blinded in its assessment of outcome data. However, the DMSC can request unblinding from the Steering Committee. Datasets on the primary outcome, SAEs and SARs will be provided by the data manager of the coordinating trial unit, Copenhagen Trial Unit.

*Closed report*

Closed report will include analysis of the safety aspects (primary outcome, SAEs, and SARs). This closed report will be prepared by the DMSC biostatistician, based on the data he obtains from the data manager of the coordinating trial unit. Only the biostatistician will have access to the anonymised microdata as provided by the coordinating trial unit.

The closed reports should provide information that is accurate. The reports should be provided to the additional DMSC members latest three days prior to the date of their online meeting.

*Minutes of the DMSC meetings*

The DMSC will prepare minutes of their meetings. The closed minutes will describe the proceedings from all DMSC meetings, including listing the DMSC’s recommendations. Because it is likely that these minutes may contain unblinded information, they must not be made available to anyone outside the DMSC.

*Open report*

Based on their closed meetings and closed reports, the DMSC will provide the SafeBoosC-IIIv Steering Committee with open reports with 1) recommendations regarding the continuation of the trial; 2) recommendations for breaking the blinding for the DMSC; and 3) if the DMSC wishes to continue the trial and their wishes to assess data the next time. This open report should not disclose the suspected intervention in the two intervention groups. The open report is only for the eyes of the Steering Committee and the contents should not be disclosed to anyone else outside the SC.

**Recommendations to the Steering Committee**

The DMSC will make a recommendation to the Steering Committee to continue, change, hold, or terminate the trial. This recommendation will be based primarily on safety considerations and will be guided by statistical monitoring guidelines, defined in this Charter. If the DMSC recommends changing, holding, or terminating the trial, an online meeting between the DMSC and the Steering Committee should be held, in which the DMSC explains its recommendation based on the dataset provided by the data manager.

If the DMSC requests breaking of the blind, the Steering Committee can ask the data manager of Copenhagen Trial Unit to inform the DMSC of the nature of the two intervention groups.

The Steering Committee is jointly responsible with the DMSC for safeguarding the interests of participants, potential future participants, and for the conduct of the trial. Recommendations to amend the protocol or conduction of the trial made by the DMSC will be considered and accepted or rejected by the Steering Committee. The Steering Committee will be responsible for deciding whether to continue, change, hold, or stop the trial based on the DMSC recommendations.

The DMSC will be notified of all changes to the trial protocol or conduct. The DMSC concurrence will be sought on all substantive recommendations, changes to the protocol, or trial conduct prior to their implementation.

**Statistical monitoring guidelines**

The statistical monitoring guidelines below are meant as a recommendation to the DMSC. However, the DMSC may use their own best judgement and modify the guidelines accordingly.

The variables below are defined in the SafeBoosC-IIIv trial protocol. For the two groups, the DMSC will evaluate data on:

- Number of participants randomised
- Number of participants randomised per intervention group (0,1)
- Number of participants stratified per stratification variable per intervention group (0,1)
- Number of hospital-free days within 90 days from randomisation per participant per intervention group (0,1)
- Number of participants registered with a specific SAE (e.g., brain injury, late-onset sepsis, bronchopulmonary dysplasia) per intervention group (0,1)
- Number of participants registered with one or more SAEs per intervention group (0,1)
- Number of participants registered with a specific SAR (e.g., severe skin injury, critical displacement of endotracheal tube) in the experimental group
- Number of participants registered with one or more SARs in the experimental group

Based on evaluations of these outcomes, the DMSC will decide if they want further data from the coordinating trial unit, and when next to perform analyses of the data.

The DMSC are recommended to use alpha spending boundaries for evaluation; we recommend Lan-DeMets sequential monitoring boundaries (applying the O’Brien-Fleming alpha-spending function). For the calculation of the sequential monitoring boundaries for benefit or harm on the primary outcome, the required sample size should be based on an assumed 5% relative risk reduction of death and an absolute increase of 3 days in the estimate of hospital-free days in surviving participants (with cerebral oximetry), at an alpha of 5% and a beta of 10%. More details on the sample size calculation for the primary outcome is available in the trial protocol, Appendix E (available on [www.safeboosc.eu](http://www.safeboosc.eu)). For the safety outcomes (SAEs and SARs), the statistical limit to guide its recommendations regarding early termination of the trial for harms, is recommended also to be conservative.

**Conditions for transfer of data from the coordinating trial unit to the DMSC**

The DMSC shall be provided with the data described below in two datasets (files), since SARs can only occur in the experimental group and thus, reporting of SARs in the file including the randomisation code, would unblind the DMSC.

The DMSC will be provided with a comma separated data file, containing the data defined as follows:

1. Row 1 contains the names of variables (to be defined below)
2. Row 2 to N (where N-1 is the number of participants who have entered the trial) each contains the data for one participant
3. Column 1 to p (where p is the number of variables defined below) each contains in row 1, the name of a variable and in the next N rows, the values of this variable

The values of the following variables should be included in the first dataset:

1. The randomisation code – the DMSC is not to be informed on what intervention the groups received
2. Site IDs blinded under a new name based on seed generation by the absolute time of the computer.
3. Gestational age ≤ or >34 weeks (stratification variable)
4. Invasive mechanical ventilation due to surgery (stratification variable)
5. Hospital-free days within 90 days from randomisation (primary outcome)
6. Death (SAE)
7. Bronchopulmonary dysplasia grade ≥2 (SAE)
8. Any brain injury diagnosed on imaging (SAE)
9. Seizures treated with antiepileptic medicine (SAE)
10. Vasoactive drugs or hydrocortisone treatment due to hypotension (SAE)
11. Vasoactive drugs or hydrocortisone treatment due to compromised circulation (SAE)
12. ExtraCorporal Membrane Oxygenation (SAE)
13. Necrotizing enterocolitis Bell’s stage≥2 or spontaneous intestinal perforation grade ≥3 (SAE)
14. Renal replacement therapy (SAE)
15. Suspected or confirmed late-onset sepsis (SAE)
16. Invasive mechanical ventilation-related infection (SAE)

The following variables will be included in the second dataset:

1. Site – IDs blinded under a new name based on seed generation by the absolute time of the computer.
2. Gestational age ≤ or >34 weeks (stratification variable)
3. Invasive mechanical ventilation due to surgery (stratification variable)
4. Severe skin damage (SAR)
5. Critical displacement of endotracheal tube threatening the life of the infant (SAR)
6. Critical displacement of endovascular line threatening the life of the infant (SAR)
7. Mismanagement while trying to improve respiratory status (SAR)
8. Mismanagement while trying to improve cardiovascular status (SAR)
9. Mismanagement while trying to improve oxygen transport (SAR)

**Anonymised data**

As no information on ‘Participant ID’ or actual ‘Site ID’ will be included in the datasets provided to the DMSC, data is considered anonymised. Furthermore, data points have been chosen according to the ‘privacy by design’ principle, i.e., only the most necessary data points will be included in the dataset and available to the DMSC.

**Listing of data points in the proposed datasets for the DMSC:**

Abbreviations:

R: Randomisation form

E: End of monitoring form

S: SAR form

F: 90-days follow-up form

***First dataset***

- R01_gestationalage: “1” is ≤34 weeks, “2” is >34 weeks
- R02_mechventsurgery: “4” is yes, “8” is no
- R03_site: This yields the blinded values (site IDs) generated by the computer
- R04_outcome: This yields the value “K” or “F”, which is coding for the randomisation outcome
- F10_totalhospfreedays (total number of hospital-free days): 0-90 days
- F01_death (death): “1” is yes, “0” is no
- F11_bpd (bronchopulmonary dysplasia grade 2 or higher): “1” is yes, “0” is no
- F12a_braininjuryimaging (brain injury on imaging): “0” is no injuries, “1” is IVH grade 2, “2” is IVH grade 3, “3” is periventricular haemorrhagic (venous) infarction, “4” is PHVD, “5” is other parenchyma haemorrhage, “6” is periventricular leukomalacia grade 2, “7” is extensive punctate white matter injury”, “8” is acute HI brain damage, “9” is cerebellar haemorrhage, “10” is stroke, “11” is big extra-axial bleeding or parenchymal bleeding/contusion, “12” is other.
- F13_seizure (seizures treated with anti-epileptic medicine): “1” is yes, “0” is no
- F14_arterialhypotension (vasoactive drugs or hydrocortisone due to hypotension): “1” is yes, “0” is no
- F15_compsystcirc (vasoactive drugs or hydrocortisone due to compromised systemtic circulation): “1” is yes, “0” is no
- F16_ecmo (ExtraCorporal Membrane Oxygenation): “1” is yes, “0” is no
- F17_boweladverseevent (necrotizing enterocolitis Bell’s stage ≥2 or spontaneous intestinal perforation grade ≥3): “1” is yes, “0” is no
- F18_rrt (renal replacement therapy): “1” is yes, “0” is no
- F19_suspectsepsis (confirmed or suspected late-onset sepsis): “1” is yes, “0” is no
- F20_ventilationfection (mechanical-ventilation related infection): “1” is yes, “0” is no

***Second dataset***

- R01_gestationalage: “1” is ≤34 weeks, “2” is >34 weeks
- R02_mechventsurgery: “4” is yes, “8” is no
- R03_site: This yields the blinded values (site IDs) generated by the computer
- SAR02_specification (type of SAR): “0” is severe skin damage, “1” is critical displacement of endotracheal tube threatening the life of the infant, “2” is critical displacement of endovascular line threatening the life of the infant, “3” is mismanagement while trying to improve respiratory status, “4” is mismanagement while trying to improve cardiovascular status, and “5” is mismanagement while trying to improve oxygen transport.

**Mapping of proposed datasets to current datasets**

***First dataset***

1. Site: the value (blinded, computer-generated site ID) of R03_site
2. Gestational age: if value in field R01_gestational age is 1, the participant has a gestational age ≤34 weeks. If value is 2, the participant has a gestational age >34 weeks
3. Invasive mechanical ventilation due to surgery: if value in field R02_mechventsurgery is 4, the participant was put on invasive mechanical ventilation due to surgery. If value is 8, the participant was put on invasive mechanical ventilation due to other reasons (‘primary pulmonary problem’ or ‘other cause)
4. The randomisation code: the value of R04_outcome
5. Hospital-free days: if value in field F10_totalhospfreedays is 0, the participant had no hospital-free days within 90 days from randomisation. If value is 1-89, the participant had such number of hospital-free days within 90 days from randomisation.
6. Death: if value in field F01_death is 1, the participant has had an event
7. Bronchopulmonary dysplasia grade ≥2: if value in field F11_bpd is 1, the participant has had an event
8. Brain injury on imaging: if value in field F12a_braininjuryimaging is 0-11, the participant has had an event
9. Seizure treated with anti-epileptic medicine: if value in field F13_seizure is 1, the participant has had an event
10. Vasopressor or hydrocortisone due to arterial hypotension: if value in field F14_arterialhypotension is 1, the participant has had an event
11. Vasopressor or hydrocortisone due to compromised systemic circulation: if value in field F15_compsystcirc is 1, the participant has had an event
12. ExtraCorporal Membrane Oxygenation: if value in field F16_ecmo is 1, the participant has had an event
13. NEC or SIP: if value in field F17_boweladverseevent is 1, the participant has had an event
14. Renal replacement therapy: if value in field F18_rrt is 1, the participant has had an event
15. Confirmed or suspected late-onset sepsis: if value in field F19_suspectsepsis is 1, the participant has had an event
16. Mechanical ventilation-related infection: if value in field F20_ventilationfection is 1, the participant has had an event
17. One or more serious adverse events: If value in field F01, F11, F12a, F13, F14, F15, F16, F17, F18, F19, or F20 is 1, the participant has had one or more serious adverse events

***Second dataset***

1. Site: the value (blinded, computer-generated site ID) of R03_site
2. Gestational age: if value in field F01_gestational age is 1, the participant has a gestational age ≤34 weeks. If value is 2, the participant has a gestational age >34 weeks
3. Invasive mechanical ventilation due to surgery: if value in field R02_mechventsurgery is 4, the participant was put on invasive mechanical ventilation due to surgery. If value is 8, the participant was put on invasive mechanical ventilation due to other reasons (‘primary pulmonary problem’ or ‘other cause)
4. Serious adverse reactions
   1. One or more serious adverse reactions: If value in field SAR02_specification is 1, 2, 3, 4, or 5, the participant has had one or more serious adverse reaction
   2. Severe skin injury: if value in field SAR02_specification is 1, the participant has had an event
   3. Critical displacement of endotracheal tube threatening the life of the baby: if value in field SAR02_specification is 2, the participant has had an event
   4. Critical displacement of endovascular line threatening the life of the baby: if value in field SAR02_specification is 3, the participant has had a an event
   5. Clinical mismanagement while trying to improve respiratory status: if value in field SAR02_specification is 4, the participant has had an event
   6. Clinical mismanagement while trying to improve cardiovascular status: if value in field SAR02_specification is 5, the participant has had an event
   7. Clinical mismanagement while trying to improve oxygen transport status: if value in field SAR02_specification

**Central data monitoring plan**

# **Purpose**

Central data monitoring for step one of the SafeBoosC-IIIv trial (90 days outcome assessment) will be conducted as outlined in this central data monitoring plan. The purpose of central data monitoring is to optimize data quality by identifying:

1. Participants with missing data
2. Sites with pre-defined quality deficiencies or noteworthy data deviations

Safety monitoring is not included in the central data monitoring plan; this task is delegated to the Data Monitoring and Safety Committee.

The central data monitoring group will include Caroline Kamp, Johanne Juul Petersen, Janus Christian Jakobsen, Markus Harboe Olsen, two board-certified neonatologists, and Mathias Lühr Hansen.

# **Blinding**

The data manager will provide two separate data sets. Data will be provided without any information about the group assignment to assess quality deficiencies and noteworthy data deviations. A second dataset with scrambled participants’ IDs will be provided to assess quality deficiencies and the variable ‘Change of medical management due to cerebral hypoxia’ (see section on ‘Noteworthy data devations’). This dataset will have information about intervention (coded as A or B). All participants from sites with fewer than five participants included will be removed from the second file.

# **Missing data**

The first part of the central data monitoring plan will focus on identifying participants with missing data entries.

## Form completeness

Each participant enrolled in the trial has two data entry forms that should be completed at a specific due date. Participants randomised to the cerebral oximetry group have a third data entry form on Serious Adverse Reactions (SARs) (**Table 1**). Since this is a pragmatic trial with limited resources and time allocated to the principal investigators, it is unrealistic to expect data entry forms to be completed at the exact due date. Therefore, a data entry form will not be flagged as ‘missing data’ until 10 days from the due date, defined as ‘overdue date’ (Table 1).

**Table 1.** Due and overdue time points for completion of data entry forms.

| **Form** | **Due** | **Overdue** |
| --- | --- | --- |
| SARs | 28 days (from birth) | 38 days (from birth) |
| End of monitoring | 28 days (from birth) | 38 days (from birth) |
| 90 days follow-up | 90 days (from randomisation) | 100 days (from randomisation) |

## Flagging of data entry forms

The ‘enrolment list’ report in the electronic case report form (eCRF) indicates the completion status of each data entry form for each participant. Once a month, the ‘enrolment list’ will be exported from the eCRF and used to assess the completion of the data entry forms for each enrolled participant. Only participants that have reached 28 days from randomisation, will be included in the data completion report since participants that have not reached this time point do not have ‘missing data’ yet, as per the definition of missing data below. Depending on the completion status, the data entry forms will be flagged as follows:

***Waiting for data entry***

A form will be marked as ‘waiting for data entry’ if the overdue date has not surpassed (e.g., the end of monitoring form for a participant 30 days after birth, will be flagged as ‘waiting for data entry’ if the form has not been completed, since the due date of 28 days from birth has surpassed – but not the overdue date of 38 days from birth).

***Missing data***

A form will be flagged as ‘missing data’ if the overdue date has surpassed (e.g., the end of monitoring form for a participant 39 days after birth will be flagged as ‘missing data’ if the form has not been completed, since the overdue date of 38 days from birth has passed).

***Completed data***

A form will be marked as ‘completed data’ if the data entry form has been completed, regardless of the previous status of the form (e.g., a form that was previously flagged as ‘missing data’ will, after the form has been completed, be flagged as ‘completed data’ like those forms that was completed in due time).

# **Quality deficiencies**

The second part of the central data monitoring will focus on identifying quality deficiencies and noteworthy data deviations.

We will aim at identifying sites with prespecified quality deficiencies as defined below:

1. Lack of cerebral oximetry monitoring during the first episode of invasive mechanical ventilation, despite being randomised to the cerebral oximetry group (E09 = No)
2. Discontinuation of cerebral oximetry monitoring for more than 12 continuous hours during the intervention period if the participant is randomised to cerebral oximetry (E10 = Yes)
3. Late initiation of cerebral oximetry monitoring in hours, defined as >6 hours from initiation of invasive mechanical ventilation if the participant is randomised to cerebral oximetry (E11 >6 hours)
4. Termination of cerebral oximetry monitoring due to other reasons than the four stopping criteria depicted in the protocol if the participant is randomised to cerebral oximetry (E15 = Other)
5. Deviation from per-protocol intervention (E09 = No OR E10 = yes OR E11>6 OR E15 = Other)
6. Visible cerebral oximetry monitoring despite being randomised to the usual care group (E17a = Yes)
7. Invalid days from birth to cerebral oximetry (E12 ≠ days from S01 to R05 (+1 day)
8. Continued consent/assent after randomisation by the parents (E18 = No OR E19 = No OR E20 = No)
9. Eligible infants not randomised, i.e., screening failures (S05 = No OR S06 = Consent not sought OR S06 = Opt-out AND S06b = No OR S06c = Yes OR S06 = prior consent AND S06a = no OR S09 = No)

## Assessment

For visualisation of each quality deficiency (except for the assessment of screening failures, see below), we will create a stacked bar chart showing the proportion of participants in the different categories for each of the variables per site. To allow further exploration of the registered quality deficiencies, the trial manager may request the IDs of participants registered with the specific quality deficiencies.

### **Assessment of screening failures**

A screening failure will be defined as a participant fulfilling the following eligibility criteria: Postnatal age <28 days (S01<28 days from actual date); GA >28 weeks (S03 = Yes); expected to receive mechanical ventilation >24 hours (S04 = Yes); no suspicion of or confirmed brain injury disorder (S07 = No); no suspicion or diagnosis of congenital heart malformation likely to require surgery (S08 = No)

And where the infant was still not randomised due to lack of adherence to the additional eligibility criteria or due to other (specified reason) as defined below:

- No possibility of initiating cerebral oximetry monitoring <6 hours from initiation of mechanical ventilation (S05 = No)
- Consent not sought (S06 = Consent not sought)
- Infants screened with opt-out and no parental information provided or parental opt-out before randomisation (S06 = Opt-out AND S06b = No OR S06c = Yes)
- Infants screened with prior consent and no parental consent (S06 = Prior consent AND S06a = No)
- Not randomised despite adhering to all eligibility criteria (S09 = 0, possibility to specify)

For visualisation, we will

1. Create a flow chart that includes the number of screened participants, the number of screening failures, including reasons, and the number of randomised participants.
2. Create a stacked bar chart showing the proportion of screening failures per site.

# **Noteworthy data deviations**

We will aim at identifying sites with noteworthy data deviations, defined as:

1. Outliers due to suspected random errors in data entries or insufficient validation ranges in the eCRF (suspected outliers)
2. Suspected systematic errors in data entries due to misunderstandings (suspected misunderstandings)
3. Potentially fabricated data (suspected fabricated data).

***Suspected outliers***

Suspected outliers of the eCRF are defined as outlying observations for continuous data points in the monitoring data report, identified by visual inspection. If such a data entry was identified in the data monitoring report, it would be marked as a suspected outlier.

***Suspected misunderstandings***

Suspected misunderstandings are defined as any unexpected differences in the distribution of data among sites, that may represent differences in coding practice or misinterpretation of the eCRF, or the overall study design. An example of this could be a local misunderstanding of the definition of a trial outcome, e.g., diagnosing the outcome by different criteria than described in the study protocol. In such a situation, we would expect an abnormally high or low frequency of the event for binary data, or a shift/abnormally large variability in the data distribution for a continuous outcome, in the site where the misunderstanding occurred.

***Suspected fabricated data***

Suspected fabricated data is defined as an unexpected distribution or variance in data at each site, that may represent fabricated data. For continuous data, we would expect to see a different shape or distribution of data when data visualised graphically, since natural variance is difficult to fabricate. An example of this could be an unexpected narrow or wide distribution of continuous variables at a particular site. An unexpectedly large or small prevalence of a binary data point can also be a sign of data fabrication or due to a misunderstanding (see suspected misunderstandings below).

## Assessment

### **Assessment of continuous variables**

We will create box plots showing the median, interquartile range, upper and lower range, and outliers for each of the following variables per site.

- Randomisation age in days and hours (time from S01 to R05)
- Gestational age at randomisation in weeks and days (E01 numeric)
- Hospital-free days based on health care records (F08 numeric)
- Parental-reported additional hospital days (F09 numeric)
- Total number of hospital-free days (F10 numeric)
- Days with invasive mechanical ventilation (F21 numeric)

### **Assessment of binary and categorical variables**

We will create a stacked bar chart showing the proportion of participants in the different categories for each of the following variables per site.

- Consent method used for randomised infants (R05 timestamp AND S06 prior informed consent, deferred consent, opt-out, or consent not sought)
- Reason for mechanical ventilation (E08 = surgery to allow for optimal pain relief, surgery for physiological/anatomical reasons, primary pulmonary problem, other)
- Birth weight <-2/>+2 SD (E01 gestational age and E02 birthweight to determine)
- Change of medical management due to cerebral hypoxia (E14 = yes)
  - Intervention categories (E14a = volume expansion, vasopressor/inotrope, PDA treatment, RBC transfusion, FiO2 adjustment, ventilator settings, other)
- Reason for termination of cerebral oximetry monitoring (E15 = stabilised cardio-pulmonary function, extubated, >28 days from birth, deceased, other)
- Death (F01 = Yes)
- Reintubation and mechanical ventilation beyond first episode (F04 = Yes)
- Bronchopulmonary dysplasia (F11 = Yes)
- Brain injury (F12 = Yes)
- Seizure (F13 = Yes)
- Vasoactive drugs or hydrocortisone due to hemodynamic insufficiency (F14 or F15 = Yes)
- ECMO (F16 = Yes)
- NEC or SIP (F17 = Yes)
- Renal replacement therapy (F18 = Yes)
- Late-onset sepsis (F19 = Yes)
- Invasive mechanical-ventilation related infection (F20 = Yes)

# **Data completion report and corrective actions**

## Missing data report

Based on the monthly data extraction from the eCRF on completion of data entry forms, a site-specific completion rate for each form (SARs, end of monitoring, 90 days follow-up) will be calculated. Data form completion will be calculated by dividing the number of forms flagged as ‘completed’ by the number of forms expected to be completed (forms that have surpassed the overdue date).

Example:

*In site DK01, 10 participants have surpassed the overdue date (>100 days from randomisation) for the 90 days follow-up form, but the form has only been completed for 7 participants.*

*90 days follow-up form completion for site DK01: 7/10*100 = 70%*

The percentage of incomplete forms for each site will be reported in a monthly data completion report. The report will be assessed and approved by the central data monitoring group and uploaded to ‘www.safeboosc.eu’.

### **Site-specific reports**

A site-specific missing data reported will automatically be generated as well. This report will include the following information:

- Completion rate for each data entry form for all sites in the trial combined
- Completion rate for each form in the specific site
- Overview of completion status for each form per participant in the specific site

To minimise missing data and loss to follow-up for the 90-day primary outcome, the site-specific report will also include a list of participant IDs for the specific site, on participants where the time to primary outcome assessment is less than 30 days, including the specific due date for primary outcome assessment (review of clinical records and contact to parents).

The trial manager will, each month, forward the site-specific report to the relevant principal investigator. As there will be zero tolerance for missing data entries, the investigators will be requested to fill out the missing data entries as soon as possible.

Missing data monitoring will start after 100 participants have been randomised. An example on the overall missing data report, and the site-specific missing data report, including the participant-specific missing data overview available in the Appendix (Figure A1, A2 and A3).

## Data quality report

Every third month, a data quality report on potential quality deficiencies and noteworthy data deviations will be generated automatically after data extraction from the eCRF. We will only include data from sites with more than five included participants and for every variable, a minimum of five participants must be added to be presented in the report. This is done to ensure some degree of anonymisation and since data assessment is difficult in very small samples. Site IDs will be blinded in the report, by assigning the sites with a new report-specific name based on a seed generated by the absolute time of the computer. Quality deficiencies or noteworthy data deviations flagged during previous central data monitoring meetings and registered in the assessment log, will be flagged in the data quality report.

The central monitoring group will assess the data quality report during a central monitoring meeting.

### **Quality deficiencies**

If a site is found to have a high number or proportion of the prespecified quality deficiencies, the trial manager will contact the relevant principal investigator for further assessment. The central monitoring group may also decide that the principal investigator should be contacted for further assessment, if only few (or a single) deficiencies have been registered. Contact despite only a few (or a single) quality deficiencies being registered could be 1) clarify if the data entry flagged as a quality deficiency is correct, and 2) discuss how such quality deficiencies can be minimised in the future.

### **Noteworthy data deviations**

Every variable will be assessed by the central monitoring group via the following steps:

1. Is there a site with noteworthy data as per the previous definitions? [Yes] / [No]
2. If yes, which suspicion has been raised? [Describe]
3. Will any course of action be taken? [Yes] / [No]
4. If yes, the trial manager will contact the principal investigator and take the relevant course of action
5. Results of the course of action will be noted

Monitoring of quality deficiencies and noteworthy data deviations will start after the first 100 participants have been randomised.

### **Assessment log**

Quality deficiencies and noteworthy data deviations, including the course of action taken (e.g., contact to principal investigator) and the summary of the course of action will be registered in the assessment log (template available in Appendix, Table A1 and A2).

### **Final data quality report**

Once the data quality monitoring report and the planned actions registered in the assessment log have been approved by the central monitoring group, a conversion key will be sent to the trial manager for re-identification of sites, in order to contact relevant principal investigators. Once the trial manager has been in touch with all relevant investigators, the report and assessment log will be updated accordingly. A short version of the final report will be uploaded to safeboosc.eu.

# **Appendix**

**Figure A1. Example of data completion report**


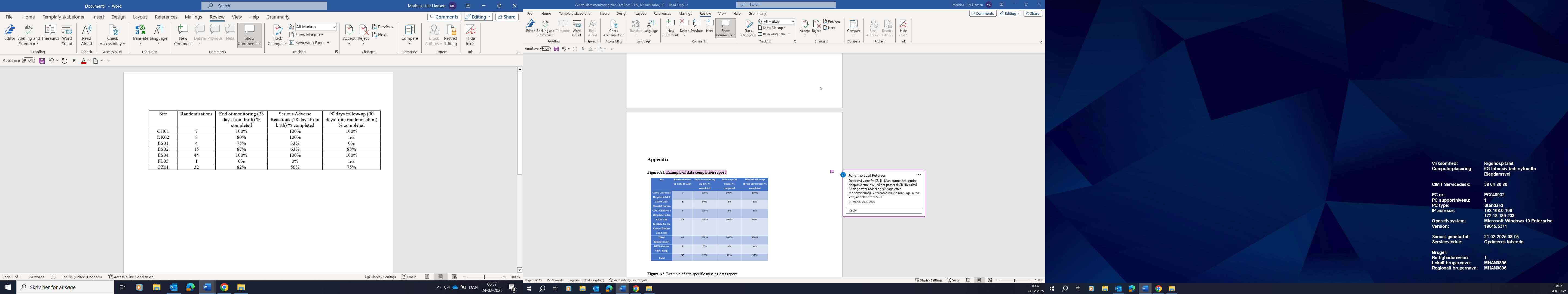


**Figure A2.** Example of site-specific missing data report


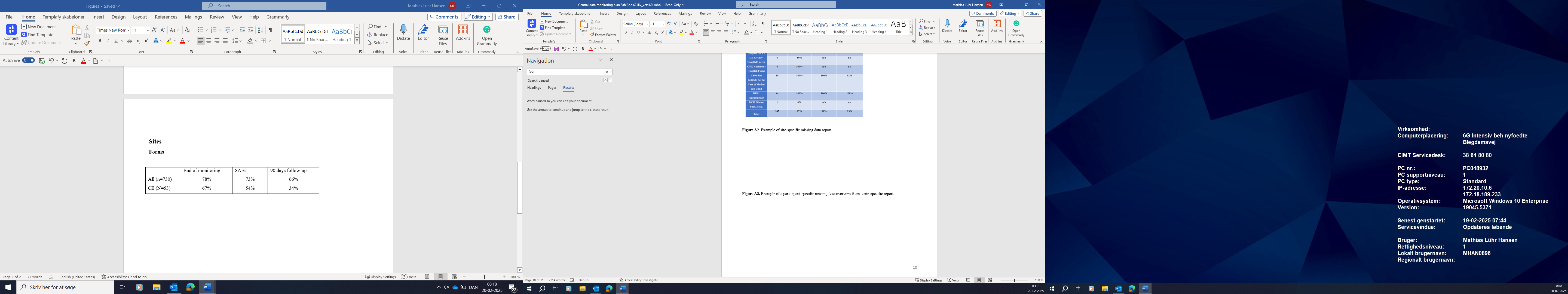


**Figure A3.** Example of a participant-specific missing data overview from a site-specific report.


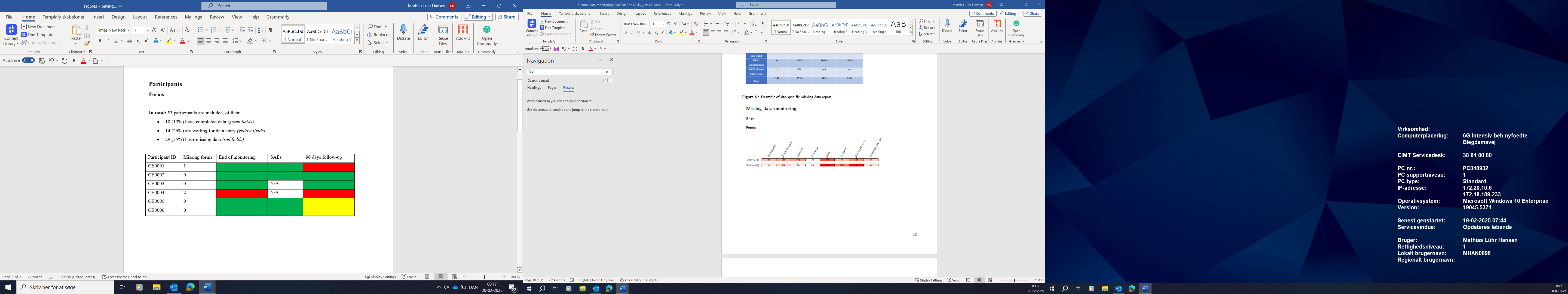


**Table A1.** Quality deficiencies on assessment log, template

| **Quality deficiency** | **Blinded site ID** | **Comment** | **Will any course of action be taken? (yes/no)** | **Unblinded site ID** | **Summary of course of action** |
| --- | --- | --- | --- | --- | --- |
|  |  |  |  |  |  |
|  |  |  |  |  |  |
|  |  |  |  |  |  |

**Table A2**. Noteworthy data deviations on assessment log, template.

| **Variable** | **Blinded site ID** | **Suspicion raised (suspected outlier, misunderstanding, or fabrication)** | **Will any course of action be taken? (yes/no)** | **Unblinded site ID** | **Summary of course of action** |
| --- | --- | --- | --- | --- | --- |
|  |  |  |  |  |  |
|  |  |  |  |  |  |
|  |  |  |  |  |  |

## World Health Organization Trial Registration Data Set (Version 1.3.1) {1b}

| **No.** | **Data Item** | **Details** |
| --- | --- | --- |
| 1 | Primary Registry and Trial Identifying Number | ClinicalTrial.gov: NCT05907317 |
| 2 | Date of Registration in Primary Registry | 8 June 2023 |
| 3 | Secondary Identifying Numbers | The Committees on Health Research Ethics in the Capital Region of Denmark ID: H-25051892 |
| 4 | Source(s) of Monetary or Material Support | Independent Research Fund Denmark |
| 5 | Primary Sponsor | Copenhagen Trial Unit, Centre for Clinical Intervention Research, Copenhagen University Hospital – Rigshospitalet, Copenhagen, Denmark |
| 6 | Secondary Sponsor(s) | N/A |
| 7 | Contact for Public Queries | Caroline Barkholt Kamp: [caroline.kamp@ctu.dk](mailto:caroline.kamp@ctu.dk), +45 3545 7171,  Copenhagen Trial Unit, Centre for Clinical Intervention Research, Copenhagen University Hospital – Rigshospitalet, Blegdamsvej 9, DK-2100 Copenhagen Ø, Denmark |
| 8 | Contact for Scientific Queries | Caroline Barkholt Kamp, Senior trial manager, PhD, Psychologist: caroline.kamp@ctu.dk, +45 3545 7171,  Copenhagen Trial Unit, Centre for Clinical Intervention Research, Copenhagen University Hospital – Rigshospitalet, Blegdamsvej 9, DK-2100 Copenhagen Ø, Denmark |
| 9 | Public Title | Treatment guided by monitoring in newborns receiving mechanical ventilation |
| 10 | Scientific Title | Safeguarding the Brain of our smallest Children – SafeBoosC-IIIv: Treatment guided by cerebral oximetry monitoring in newborns receiving invasive mechanical ventilation. |
| 11 | Countries of Recruitment | Argentina, Australia, Austra, Belgium, Brazil, Canada, Chile, China, the Czech Republic, Denmark, Germany, Greece, India, Ireland, Italy, Poland, Romania, Spain, Switzerland, Türkiye, the United States, and Zambia |
| 12 | Health Condition(s) or Problem(s) Studied | Newborns receiving invasive mechanical ventilation |
| 13 | Intervention(s) | Treatment guided by cerebral oximetry monitoring added to usual care versus usual care |
| 14 | Key Inclusion and Exclusion Criteria | Inclusion criteria: newborns with gestational age more than or equal to 28+0; postnatal age less than 28 days, expected to receive invasive mechanical ventilation (intubation) for at least 24 hours, as judged by the physician intending to randomise, parental informed consent, unless the centre has chosen to use ‘opt-out’ or deferred consent as consent method, a cerebral oximeter available so monitoring can be started within six hours after initiation of invasive mechanical ventilation.  Exclusion criteria: suspicion of or confirmed brain injury or disorder (e.g. severe hypoxic-ischemic encephalopathy, intraventricular haemorrhage grade 3 or 4, cerebral malformation, genetic or metabolic disease), suspicion or diagnosis of congenital heart malformations likely to require surgery. |
| 15 | Study Type | An investigator-initiated, multinational, randomised, parallel, pragmatic, superiority phase III clinical trial.  The statisticians, trial manager, sponsor, writers of the two final abstracts before unblinding of the groups, and assessors of the primary outcome will be blinded. Web-based randomisation will be centrally performed. The block sizes will vary, be computer-generated, and concealed for all investigators.  We aim to assess whether treatment guided by cerebral oximetry monitoring can increase the number of hospital-free days within 90 days of randomisation in 1,610 newborns receiving invasive mechanical ventilation. |
| 16 | Date of First Enrollment | The first participant was randomised on 11 April 2025. |
| 17 | Sample Size | 1610 participants (152 currently randomised). |
| 18 | Recruitment Status | Recruiting |
| 19 | Primary Outcome(s) | Hospital-free days within 90 days of randomisation |
| 20 | Key Secondary Outcomes | Proportion of participants with one or more serious adverse events within 90 days of randomisation.  Invasive mechanical ventilation-free days within 90 days of randomisation. |
| 21 | Ethics Review | Approved on 11 December 2025 by The Committees on Health Research Ethics in the Capital Region of Denmark.  Phone: +4538666395  Email address: vek@regionh.dk |
| 22 | Completion Date | N/A |
| 23 | Summary Results | N/A |
| 24 | IPD Sharing Statement | Six months after the acceptance of the publication that presents the primary outcome, the dataset can be shared with other researchers. Before sharing, subject study numbers, will be removed, neonatal intensive care unit numbers will be replaced, gender and twin status removed, birth weight and gestational age recoded into binary variables to minimise the risk of reidentification. Use by other researchers will depend on the permission of the trial Steering Committee. |
